# The antimicrobial activity of propolis extract on Klebsiella pneumoniae and Escherichia coli strains isolated from Qazvin hospital personnel

**DOI:** 10.1099/jmm.0.002030

**Published:** 2025-08-11

**Authors:** Fatemeh Rahmani, Niloofar Kiaheyrati, Mohadeseh Khakpour, Farhad Nikkhahi, Fatemeh Fardsanei, Saeed Sayfzadeh, Mahtab Alambeigi

**Affiliations:** 1 Medical Microbiology Research Center, Qazvin University of Medical Sciences, Qazvin, Iran; 2 Department of Agronomy, Islamic Azad University Takestan, Takestan, Iran

**Keywords:** AmpC, carriers, extended-spectrum beta-lactamase (ESBL), multidrug-resistant (MDR), nosocomial infection, propolis

## Abstract

**Introduction.** Nosocomial infections, particularly those caused by Gram-negative bacilli, indeed pose significant challenges in healthcare settings. Hospital staff can act as carriers of these infections, potentially transmitting them to patients and colleagues. Propolis, a natural resinous substance collected by honeybees, has shown promising antibacterial properties against various microorganisms, including Gram-negative bacteria like *Escherichia coli* and *Klebsiella pneumoniae*.

**Hypothesis/Gap Statement.** Despite the documented antibacterial properties of propolis, limited research has evaluated its efficacy against clinical isolates from healthcare workers, particularly in Iran.

**Aim.** To evaluate the *in vitro* effect of propolis on *K. pneumoniae* and *E. coli* isolated from the nose and nails of hospital personnel in Qazvin.

**Methodology.** Fifty Gram-negative bacilli were isolated from the nose and nails of hospital personnel in Qazvin. An antibiotic sensitivity test was conducted using the disk diffusion method based on CLSI 2024 guidelines for various antibiotics. The most common isolated strain was analysed using enterobacterial repetitive consensus PCR (ERIC-PCR). Finally, the microbroth dilution method was used to assess the antibacterial effect of propolis on the isolated strains.

**Results.** The most frequent pathogens were *K. pneumoniae* (66%) followed by *E. coli* (34%). Most of the isolates were sensitive to the majority of antibiotics tested, and the highest antibiotic resistance was observed in trimethoprim/sulfamethoxazole (55%), ceftazidime (32%) and tetracycline (26%). Extended-spectrum beta-lactamase production was found in 10% of isolates of all Gram-negative bacteria. Additionally, 24% of the strains were multidrug-resistant. ERIC-PCR analysis revealed high genetic diversity among *K. pneumoniae* strains, which were the most common strains isolated from personnel. The MIC of propolis for both *K. pneumoniae* and *E. coli* was 5%, and the minimum bactericidal concentration was 10% after culturing 100 µl on Mueller–Hinton agar.

**Conclusion.** The present study showed that the isolates from the nose and nails of hospital personnel may pose a serious issue in the field of public health. These findings suggest that Iranian bee propolis has medicinal value as a natural product and was identified as an antimicrobial substance with positive effects on bacterial strains isolated from hospital personnel.

## Introduction

Infections that manifest in patients 48–72 h post-hospitalization are classified as nosocomial infections. Morbidity and mortality rates associated with hospitalization have steadily increased due to these infections, which are a significant issue for the healthcare system [1]. The most hazardous pathogens responsible for these infections include the ESKAPE pathogens (*Enterococcus faecium*, *Staphylococcus aureus*, *Klebsiella pneumoniae*, *Acinetobacter baumannii*, *Pseudomonas aeruginosa* and *Enterobacter* spp.) [2].

Nosocomial infections are attributed to two primary sources, namely, (a) infections acquired by hospitalized patients from the hospital environment and (b) infections transmitted to patients by individuals who come to the hospital or work within its premises. According to studies, almost half of all hospital infections are in the first batch. Healthcare-associated infections are classified into several categories, including catheter-associated urinary tract infections, central line-associated bloodstream infections, ventilator-associated pneumonia, surgical site infections and hospital-acquired *Clostridioides difficile* infections [3,4]. These infections can occur during healthcare delivery for other diseases and even after the discharge of the patients. Furthermore, they include occupational infections among healthcare personnel. These infections are related to invasive devices used in modern healthcare, such as catheters and ventilators.

An increase in infections is associated with a rise in long-term disability, extended hospital stays, antibiotic resistance, socioeconomic disruption and death rates [5]. Recently, the number of drug-resistant genotypes has steadily increased, and ‘superbugs’ have also arisen. This has resulted in a challenging situation for the already complex resistance mechanism and limited treatment options. Consequently, it has become even more imperative to investigate novel strategies for the treatment of drug-resistant bacteria [6,7].

In this scope, natural products and green chemistry with antimicrobial properties are gaining attention in this area. Specifically, propolis is an example of this category of agent, and it is manufactured by extracting the balsamic secretions from the flowers, shells, barks and buds of a wide variety of plant species. Honeybees extract and transform this viscous material, with the aid of their salivary secretions and beeswax, into propolis [8]. Propolis is a significant antimicrobial bee product. It exerts its effect on both Gram-positive and Gram-negative bacteria, as well as on aerobic and anaerobic strains. The potency of propolis varies depending on its chemical composition, which can differ significantly across countries. Its antibacterial activity should be considered on two levels: first, its direct action against microorganisms and, second, its ability to stimulate the immune system, thereby activating the body’s innate defence mechanisms [9,11].

So, the aim of this study was to evaluate the effect of propolis on Gram-negative bacilli isolated from the nose and nails of Qazvin hospital personnel *in vitro.* This study also sought to investigate the genetic relationships between the dominant strains identified by enterobacterial repetitive consensus PCR (ERIC-PCR).

## Methods

### Sampling

This cross-sectional study was conducted at Velayat Hospital in Qazvin, Iran, from April to September 2024. A total of 100 samples were obtained from 50 healthcare personnel from 4 different wards [operating room, intensive care unit (ICU), emergency and internal medicine unit] during a 3-month period. 50 nasal swab samplings and 50 swab samples under the fingernails from both hands of each subject were collected using sterile-moistened cotton-tipped swabs and placed into a sterile test tube.

### Bacterial isolation and identification

Sampling swabs were kept in trypticase soy broth medium, transferred to the medical microbiology laboratory, and incubated for 24 h at 37 °C. The samples were cultured on blood agar, Eosin Methylene Blue agar (EMB), MacConkey agar, and cetrimide agar to isolate a diverse range of microorganisms. Then, additional biochemical tests, such as oxidase, catalase, urease, carbohydrate utilization and motility tests, and staining techniques, such as Gram staining, were used [12].

### Antimicrobial susceptibility testing

Antibiotic susceptibility tests for Gram-negative bacilli isolates were performed using the Kirby–Bauer disk diffusion method, according to the Clinical and Laboratory Standards Institute (CLSI) 2024 guidelines. Cefotaxime (30 µg), Cefotaxime/clavulanic acid (30 µg), Ceftazidime (30 µg), Ceftazidime/clavulanic acid (30 µg), Cefoxitin (30 µg), Cefepime (30 µg), Meropenem (10 µg), Tetracycline (30 µg), Levofloxacin (5 µg), Gentamicin (10 µg) and Trimethoprim-sulfamethoxazole (25 µg) were used in this study. The isolates were categorized as resistant, intermediate and susceptible based on zone of inhibition in accordance with CLSI 2024. Colistin sensitivity was also determined using the colistin broth dilution elution method according to CLSI guidelines. Isolates that displayed resistance to three or more antimicrobial classes were deemed as multidrug-resistant (MDR). The combined test method was used to identify extended-spectrum beta-lactamase (ESBL)/AmpC-producing strains [13].

### Detection of *β*-lactamase-encoding genes

To identify ESBL genes (*bla*_TEM_, *bla*_SHV_ and *bla*_CTX-M_), AmpC-encoding genes (*bla*_ACC_, *bla*_CIT_, *bla*_EBC_, *bla*_FOX_, *bla*_MOX_ and *bla*_DHA_) and carbapenemase genes (*bla*_VIM_, *bla*_NDM_, *bla*_KPC_, *bla*_OXA-48_ and *bla*_IMP_), PCR was performed on phenotypically verified ESBL- and AmpC-positive isolates using the primers indicated in Table 1 [14,16]. The boiling method was used to extract genomic DNA. Briefly, the isolates were incubated at 37 °C for 20 h after being cultured on trypticase soy agar (TSA). Two colonies were selected and inoculated into 400 µl of Tris-EDTA buffer, heated at 100 °C for 10 min and then cooled down on ice for 15 min. Genomic DNA for the PCR test was extracted from the supernatant after centrifugation at 10,000 r.p.m. of the tube [17].

**Table 1.** Primer sequences of beta-lactamase-resistant genes

Gene	Sequence	Size (bp)
*bla*_SHV_	F-ATCCACTATCGCCAGCAG R-CCTCATTCAGTTCCGTTTCC	232
*bla*_CTX-M_	F-AGGAAGTGTGCCGCTGTATG R-CTGTCGCCCAATGCTTTACC	532
*bla*_TEM-1_	F-TCGCCGCATACACTATTCTC R-AACTTTATCCGCCTCCATCC	353
*bla*_NDM-1_	F-CAGCGCAGCTTGTCG R-TCGCGAAGCTGAGCA	233
*bla*_IMP_	F-GAATAGAATGGTTAACTCTC R-CCAAACCACTAGGTTATC	188
*bla*_VIM_	F-GTTTGGTCGCATATCGCAAC R-AATGCGCAGCACCAGGATAG	382
*bla*_KPC_	F-ATGTCACTGTATCGCCGTCTAGT R-CGTTGACGCCCAATCC	410
*bla*_OXA-48_	F-GCGTGGTTAAGGATGAACAC R-CATCAAGTTCAACCCAACCG	438
*bla*_FOX_	F-AACATGGGGTATCAGGGAGATG R-CAAAGCGCGTAACCGGATTGG	190
*bla*_MOX_	F-GCTGCTCAAGGAGCACAGGAT R-CACATTGACATAGGTGTGGTGC	520
*bla*_*DHA*_	F-TGGCCAGAACTGACAGGCAAA R-TTTCTCCTGAACGTGGCTGGC	405
*bla*_CIT_	F-TGGCCAGAACTGACAGGCAAA R-TTTCTCCTGAACGTGGCTGGC	462
*bla*_EBC_	F-TCGGTAAAGCCGATGTTGCGG R-CTTCCACTGCGGCTGCCAGTT	302
*bla*_ACC_	F-AACAGCCTCAGCAGCCGGTTA R-TTCGCCGCAATCATCCCTAGC	346

bla, β-lactamase encoding gene; F, Forward primer; R, Reverse primer.

### Molecular typing

ERIC-PCR is often used to analyse the diversity of enteric bacteria. The ERIC-PCR technique was carried out using enterobacterial repetitive consensus (ERIC) primers – ERIC1 : 5ʹ-ATGTAACGTCCTGGGGATTCAC-3ʹ and ERIC2 : 5ʹ- AAGTAAGTGACTGGGGTGAGCG-3ʹ. PCR conditions were as follows: pre-denaturation at 94 °C for 2.5 min, denaturation at 94 °C for 30 s, annealing at 47 °C for 1 min, extension at 72 °C for 1 min and post-extension at 72 °C for 4 min, which was performed in 35 cycles. PCR products were electrophoresed using 1.4% agarose gel electrophoresis stained with safe stain. ERIC profiles were compared using the Dice similarity matrix coefficient and using GelClust software to prepare the phylogenetic tree [18].

### Preparation of propolis extract

**Fig. 1.**
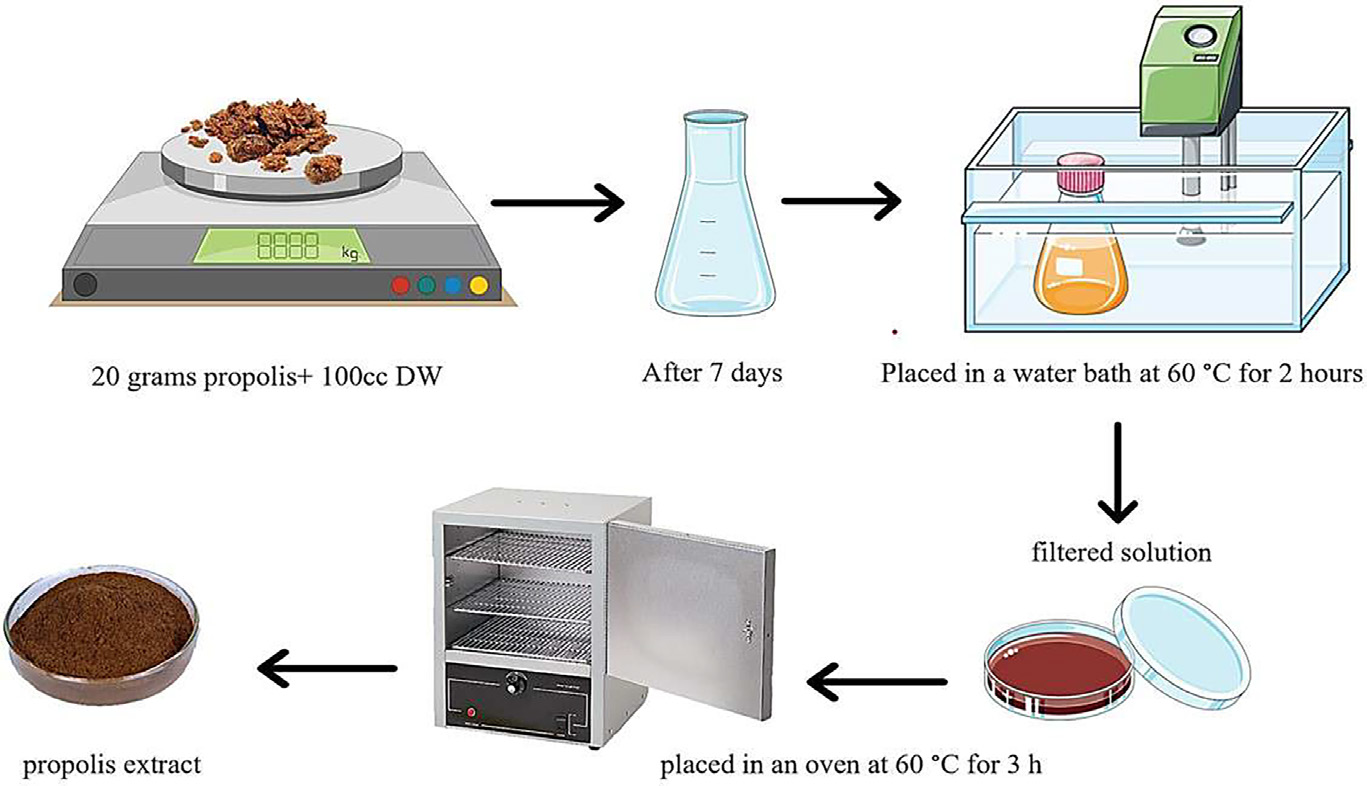
Preparation of propolis extract.

To evaluate the effect of propolis on the isolated bacterial strains, first, fresh propolis samples were prepared from bee farms around the city of Qazvin (Eastern Alamut) and transported to the laboratory under hygienic conditions and in closed sterile containers. To make the aqueous extract (Fig. 1), 20 g of local and commercial propolis was measured individually on a digital scale and placed in a sterile container with 100 ml of distilled water. The prepared solution was maintained for 7 days at room temperature and away from light. After a week, the propolis container was submerged in a water bath at 60 °C for 2 h to remove the active components that cannot be extracted with water solvent. After this period, the main propolis extract was released from its wax. The supernatant solution was filtered to remove contaminants and any remaining propolis from which the active components had been removed. The filtered solution was transferred to a sterile glass plate, which was then placed in an oven at 60 °C to concentrate and eliminate the solvent. The concentrated extract was scraped from the bottom of the plate into a powder, transforming from a liquid to a solid, after the solvent had evaporated after 3 h. This solid can be preserved at −20 °C for an extended period [19].

### Antibacterial activity assessments

#### Determination of MIC

This procedure requires diluting propolis using a serial dilution technique. We prepared bacterial suspensions with turbidity equivalent to 0.5 McFarland standard. In a sterile 96-well microplate, columns were designated for extract dilutions, while each row was allocated for various tested strains. In the first row of all columns, we added 100 µl of 0.5 McFarland bacterial suspension. Then, we added 100 µl of each of the dilutions prepared from the propolis extract to our special column. In the eighth column, we added the liquid medium along with the extract as a negative control, and in the ninth column, we added 100 µl of 0.5 McFarland bacterial suspension as a positive control. We repeated this process for each of the bacteria and finally, after finishing the setup, placed it in an incubator at 35–37 °C for 18 to 24 h. Following an incubation period of 18 to 24 h, the MIC of propolis against the strain was assessed; the first well in each row of the 96-well plate exhibiting no turbidity was designated as the MIC of propolis.

#### Determination of minimum bactericidal concentration

To determine the minimum bactericidal concentration (MBC) of propolis, we removed 100 µl from the wells in which turbidity did not occur in sterile conditions and inoculated it into TSA medium and used a Pasteur pipette with a tilted head to spread it. Then, the plates were incubated at 37 °C for 24 hours. Subsequently, the concentration capable of eliminating 99.99% of bacteria was deemed equivalent to the MBC.

## Data analysis

A checklist was used to record data. After collecting the data, the findings were presented in the form of statistical tables, graphs and numerical indicators. SPSS version 16 software was used for data analysis.

## Results

### Bacterial isolation and identification

Of the total 100 subjects examined, 50% (*n*=50) showed positive culture for different bacterial species from their fingernails and noses. *K. pneumoniae* was the predominant bacterial species (66%, *n*=33) followed by *E.coli* (34%, *n*=17). Personnel’s hands were the most contaminated part, considering both the numbers and diversity of isolated organisms. Contamination of the hospital personnel has a main role in the dispersion of nosocomial pathogens in the hospital environment. The colony isolates of *K. pneumoniae* were pink mucoid on MacConkey agar, non-motile, rod-shaped and Gram-negative. Based on biochemical tests, the isolates of *K. pneumoniae* were positive on Voges–Proskauer, citrate, urea and lysine decarboxylase tests and negative on indole and methyl red tests.

### Antimicrobial susceptibility testing

**Fig. 2.**
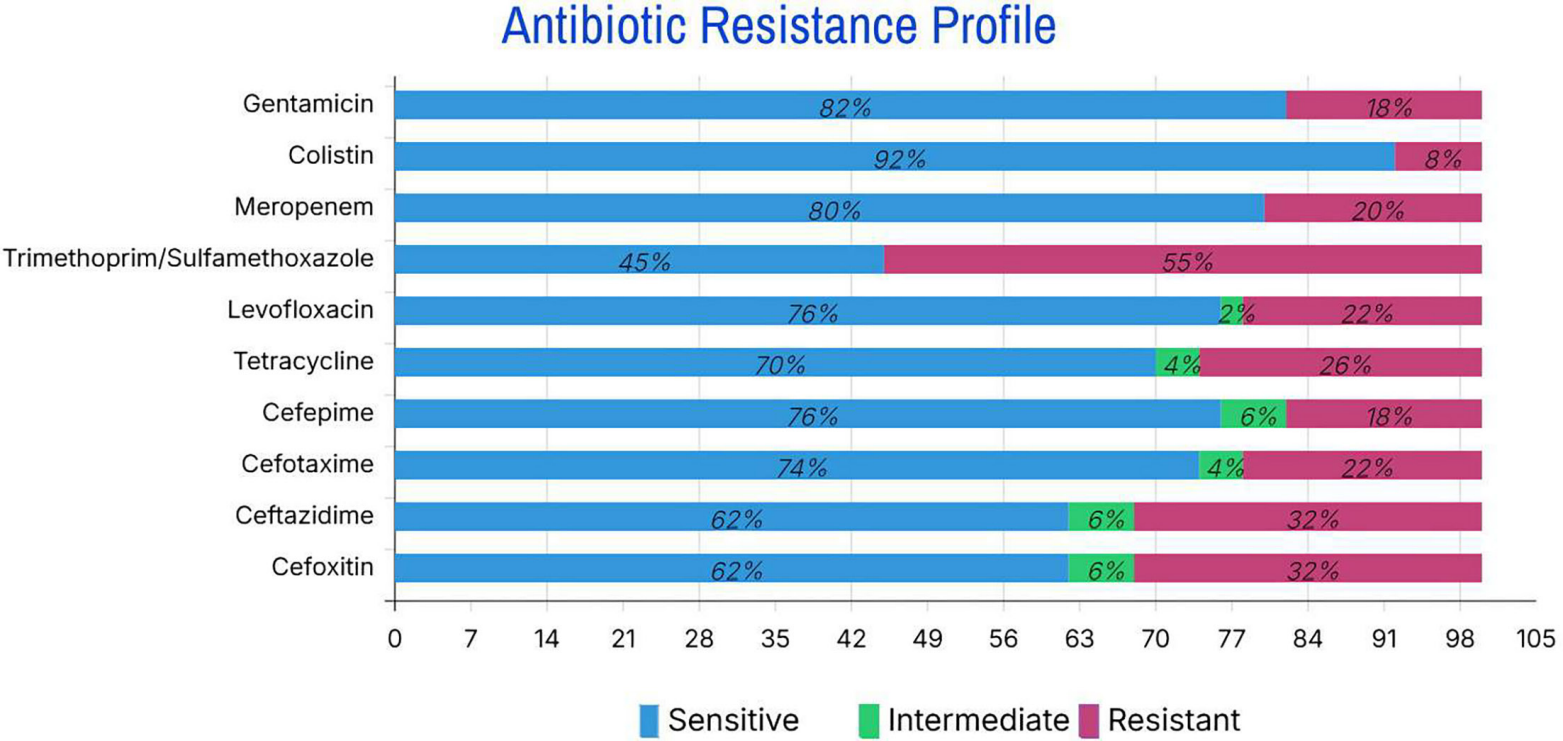
Antibiotic resistance in Gram-negative bacilli.

Based on the results of the antibiogram test, which is shown in Fig. 2, out of a total of 50 isolates, most of them were sensitive, and the highest antibiotic resistance was observed in trimethoprim/sulfamethoxazole (55%), ceftazidime (32%) and tetracycline (26%). Colistin showed the highest sensitivity (92%); also, gentamicin and meropenem showed the highest inhibitory effect, with sensitivity above 80%. Among them, 24% of the strains were MDR. 12% of the strains are phenotypically AmpC producers, and 10% of the strains are ESBL producers.

### Detection of *β*-lactamase-encoding genes

Six (12 %) strains produced AmpC, and all strains had *bla*_DHA_ and *bla*_CIT_ genes. Also, out of five (10%) strains producing ESBL, all of them harboured *bla*_TEM_ and *bla*_SHV_ genes. Also, among the 50 Gram-negative bacilli identified, 10 (20%), by using the disc diffusion method, were shown to be resistant to carbapenems. The most common genes identified were *bla*_IMP_ (100%) and *bla*_OXA-48_ (80%), whereas *bla*_VIM_, *bla*_NDM_ and *bla*_KPC_ genes were not observed in any of the isolates.

### Molecular typing of *K. pneumoniae*

**Fig. 3.**
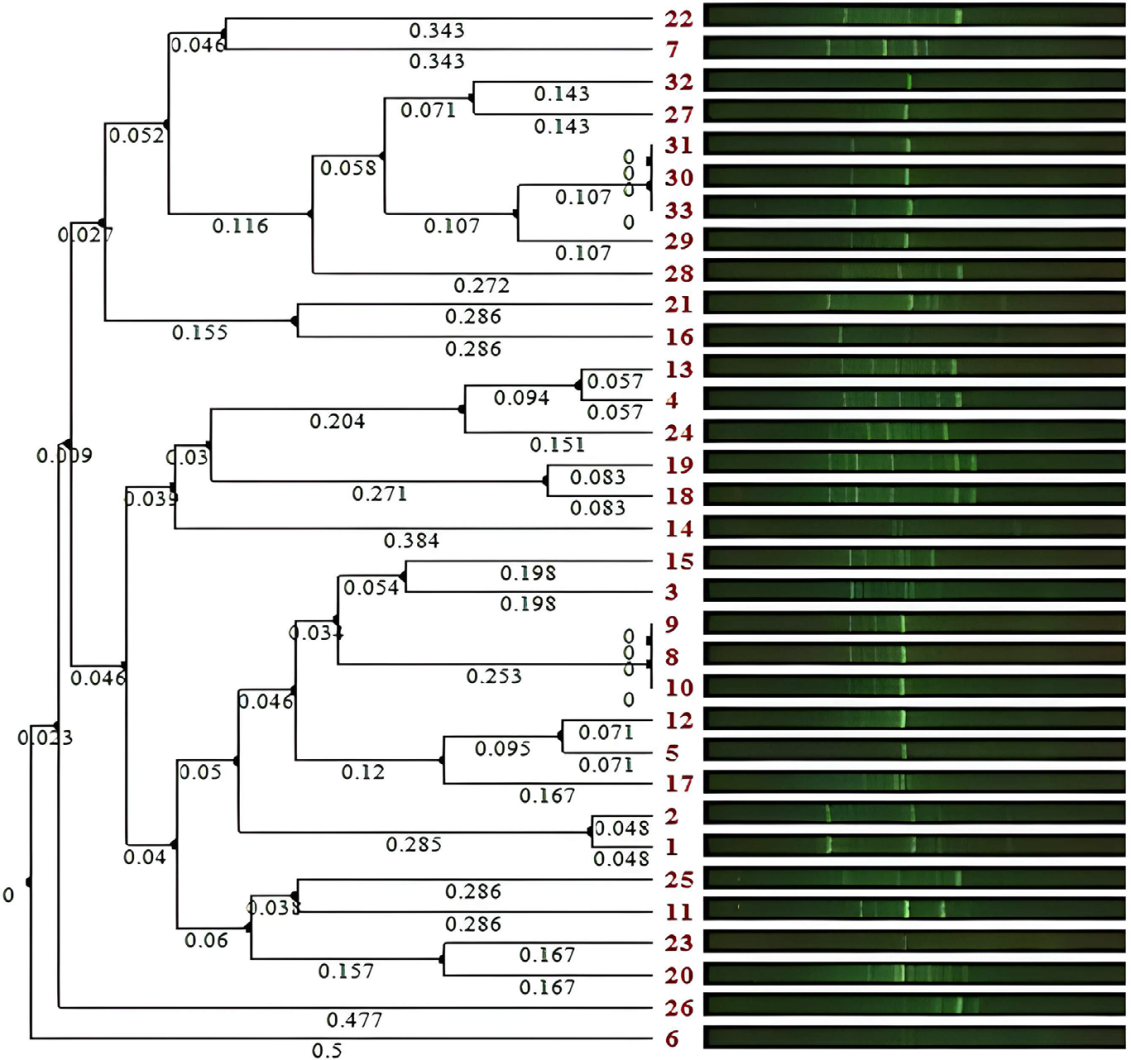
Dendrogram cluster analysis of ERIC-PCR for 33 *K. pneumoniae* strains.

The identified strains of *K. pneumoniae* were typed using the ERIC-PCR test. ERIC profiles were compared using the Dice method and clustered by the unweighted pair group method with arithmetic mean (UPGMA) program. GelClust software [18] was used to process the fingerprints, which generated a dendrogram for interpreting genetic relatedness (Fig. 3). The studied isolates had a band size ranging from 100 bp to more than 1.5 kb. A total of 24 different ERIC profiles (E-types) were observed with 80% similarity, 6 common types and 18 single types. Two common types include three isolates, which also showed similar antibiotic resistance patterns, and other isolates showed a unique pattern. Overall, a high level of genetic diversity was observed among *K. pneumoniae* strains.

### Antibacterial activity assessments

**Fig. 4.**
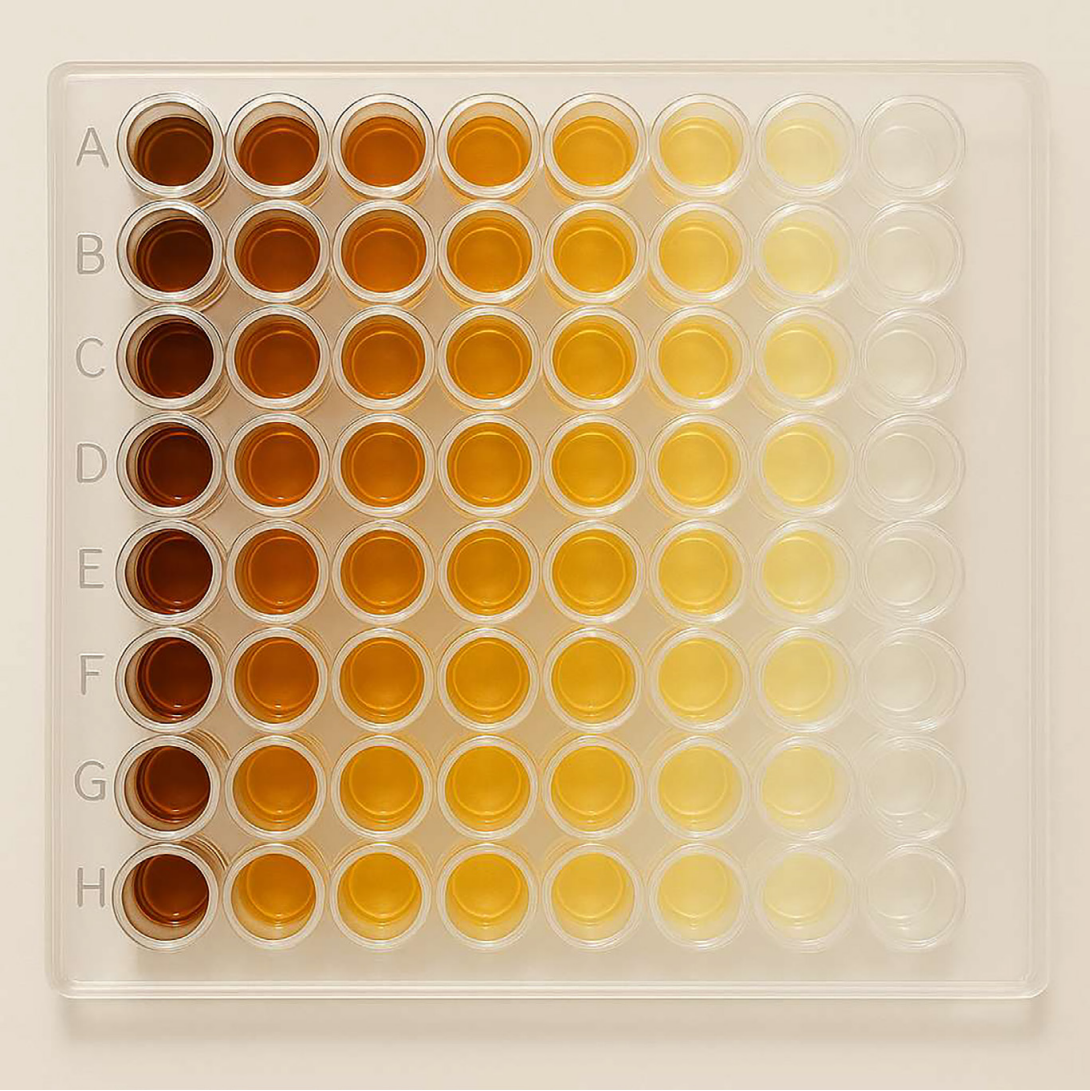
MIC of propolis against *K. pneumoniae* and *E. coli*.

MIC was determined as the lowest concentration of propolis extract that prevented the growth of the tested microorganisms. The result of propolis MIC on both strains of *K. pneumoniae* and *E. coli* visually was 5 % (50 mg ml^−1^), and MBC was considered as the minimum concentration of propolis extract that has the property of killing microorganisms, which was equal to 10% (100 mg ml^−1^) after culturing 100 µl on Mueller–Hinton agar, as shown in Fig. 4 and Table 2.

**Table 2.** MIC and MBC values of propolis extracts against Klebsiella pneumoniae and *Escherichia coli*

Propolis(mg/ml)	*Klebsiella pneumoniae*		*Escherichia coli*	
	MIC	MBC	MIC	MBC
1.5(0.1%)	+ve	+ve	+ve	+ve
3(0.3 %)	+ve	+ve	+ve	+ve
6(0.6 %)	+ve	+ve	+ve	+ve
12.5(1.25 %)	+ve	+ve	+ve	+ve
25(2.5 %)	+ve	+ve	+ve	+ve
50(5 %)	-ve	+ve	-ve	+ve
100(10 %)	-ve	-ve	-ve	-ve

## Discussion

Gram-negative bacilli are one of the main causes of hospital-acquired infections. *K. pneumoniae* is associated with urinary tract infections, pneumonia, wound infections and bloodstream infections. Following a *Klebsiella* nosocomial infection, we are also seeing an increase in the prevalence of this type of infection with *E. coli*. Since these bacteria are not ordinarily found on the hands or in the nose, the presence of these bacteria can be an indication of faecal contamination that is caused by intestinal infections.

Conversely, they are the most concerning MDR pathogens, resulting in numerous therapeutic difficulties. Hence, this study aimed to assess the impact of propolis on *K. pneumoniae* and *E. coli* strains isolated from the nails and nasal passages of hospital staff in Qazvin, Iran, under *in vitro* conditions. The findings of this investigation indicated that *K. pneumoniae* is the predominant strain identified from carriers, with a prevalence of 66%, which is in line with the results obtained from the study conducted by Liu *et al*. in China [20]. Considering the critical role of *K. pneumoniae* in nosocomial infections, genotyping clinical isolates is advantageous for the prevention of hospital-acquired infections and the identification of infection sources. A high level of genetic diversity was observed among *K. pneumoniae* strains. However, some common types were also identified. Similarly, in a study by Baraka *et al*., ERIC-PCR revealed limited genetic association between * K. pneumoniae* strains [21].

Total bacterial carriage in hand and nose swabs was 48% and 52%, respectively. The antibiogram test results from 50 isolates indicated the highest antibiotic resistance to cotrimoxazole at 55% and to ceftazidime at 32%. Aminoglycosides exhibited the greatest sensitivity. Antibiotic resistance is presently the primary focus of concern for researchers and medical professionals. Resistance in Gram-negative bacteria is mainly through the production of ESBLs, AmpC beta-lactamases and carbapenemases. The genes encoding these enzymes are often located on plasmids carrying resistance genes to other common antibiotics in clinical settings. In this study, 12% of the strains were AmpC and 10% were ESBL, which is contrary to the results of Kazemian *et al*.’s study [22], where 18% of the strains were AmpC and 43.3% of the strains were ESBL. These variances may be attributed to differences in sample size and population. Carbapenems are frequently utilized as an efficacious therapy for MDR *Enterobacteriaceae* infections.

The current analysis indicates that 20% of the strains resist meropenem. Additionally, colistin has a good sensitivity (92%) as a last resort. However, if it is used improperly and indiscriminately, colistin resistance will increase in the not-too-distant future. In Niazadeh *et al*.’s study [23], the mechanism of colistin resistance was examined among *K. pneumoniae* isolates isolated from patients with urinary tract infections hospitalized in Qazvin hospitals’ ICU departments, and 9% of the isolates were colistin resistant. Additionally, a study by Fariba Karamet in 2017 aimed at examining antibiotic resistance to colistin in hospital infections caused by multidrug-resistant *Acinetobacter* found that only 3.13% of the strains exhibited resistance to colistin, which is similar to the findings derived from our research [24].

On the other hand, as mentioned earlier, hospital personnel as carriers of infection play an important role in the spread of hospital infections. Due to the frequent contact of personnel with patients and the hospital environment, this issue can lead to the transmission of pathogenic bacteria and viruses, which can be due to non-observance of hand hygiene, incorrect use of personal protective equipment and contamination of clothes and medical equipment.

With the rise of antibiotic resistance, the scientific community is seeking alternative methods to control hospital infections. Several studies have investigated the use and effects of traditional medicines for various diseases, including the effects of herbal medicines on infectious diseases. Propolis is an antimicrobial bee product that works against both Gram-positive and Gram-negative bacteria, as well as against aerobic and anaerobic bacteria. The activity of propolis depends on the chemical composition and can vary in different countries [25]. The current investigation demonstrated a substantial antibacterial effect. The effect was discovered to be concentration-dependent, with higher propolis concentrations resulting in a considerable reduction in bacterial growth rate.

The results indicate that the MIC of propolis against *K. pneumoniae* and *E. coli* strains was 5%, while the MBC was 10%, which aligns with the study conducted by Mohammad Javad Abedi et al. in Iran in 2017 [26]. Conversely, the research by Anibijuwon *et al*. [27] demonstrated that an ethanol extract of propolis at a concentration of 12.5 (mg ml^−1^) inhibits the growth of *S. aureus, P. aeruginosa* and *K. pneumoniae*, while a concentration of 50 (mg ml^−1^) effectively eradicates the bacteria. Research discrepancies may be attributable to factors such as different sample sizes, bacterial genetic composition and antimicrobial susceptibility. Surprisingly, the antibacterial potential of propolis varies with its geographical location. Middle Eastern propolis exhibits superior efficacy against Gram-positive (*S. aureus*) and Gram-negative (*E. coli*) pathogens. In contrast, German, Irish and Korean propolis exhibit the lowest activity [10].

## Conclusion

The present study showed that the isolates from the nose and nails of hospital personnel pose a serious issue in the field of public health. The growing resistance of these bacteria indicates potential risks for the spread of resistant infections in society, which requires careful monitoring of hospital personnel’s health. As a result, due to the small sample size, the study demonstrated less prevalence; hence, an increase in sample size can be considered for the future scope of the study. According to the results obtained from this research, it can be concluded that the ERIC-PCR method is a suitable method for molecular typing of isolated strains and identifying infection sources. Also, propolis was identified as an antimicrobial agent with positive effects on bacterial strains isolated from hospital personnel. These results can serve as a basis for further research on the use of propolis in controlling hospital infections and improving patient safety.

## Abbreviations

CLSI: Clinical and Laboratory Standards Institute
ERIC: enterobacterial repetitive consensus
ERIC-PCR: enterobacterial repetitive consensus PCR
ICU: intensive care unit
MBC: minimum bactericidal concentration
TSA: trypticase soy agar
